# The Role of Endometrial Sampling before Hysterectomy in Premenopausal Women with Abnormal Uterine Bleeding

**DOI:** 10.3390/jcm13133709

**Published:** 2024-06-25

**Authors:** Oguzhan Kuru, Ipek Betul Ozcivit Erkan, Cansu Turker Saricoban, Utku Akgor, Neslihan Gokmen Inan, Sennur Ilvan

**Affiliations:** 1 Division of Gynecologic Oncology, Department of Obstetrics and Gynecology, Cerrahpasa Faculty of Medicine, Istanbul University-Cerrahpasa, Istanbul 34098, Türkiye; oguzhan.kuru@iuc.edu.tr; 2 Department of Obstetrics and Gynecology, Cerrahpasa Faculty of Medicine, Istanbul University-Cerrahpasa, Istanbul 34098, Türkiye; 3 Department of Pathology, Cerrahpasa Faculty of Medicine, Istanbul University-Cerrahpasa, Istanbul 34098, Türkiye; cansu.saricoban@iuc.edu.tr (C.T.S.); ilvan@iuc.edu.tr (S.I.); 4 Division of Gynecologic Oncology, Department of Obstetrics and Gynecology, Hacettepe University Faculty of Medicine, Ankara 06230, Türkiye; utkuakgor@hacettepe.edu.tr; 5 Department of Computer Engineering, College of Engineering, Koc University, Istanbul 34450, Türkiye; ninan@ku.edu.tr

**Keywords:** abnormal uterine bleeding, dysfunctional uterine bleeding, endometrial neoplasms, endometrial biopsy

## Abstract

**Background/Objectives:** An endometrial sampling is recommended for patients experiencing abnormal uterine bleeding above the age of 40 or 45. Valid risk prediction models are needed to accurately assess the risk of endometrial cancer and avoid an unnecessary endometrial biopsy in premenopausal women. We aimed to assess the necessity and usefulness of preoperative endometrial sampling by evaluating premenopausal women who underwent hysterectomy for abnormal uterine bleeding after preoperative endometrial sampling at our clinic. **Methods:** A retrospective analysis was conducted on 339 patients who underwent preoperative endometrial sampling and subsequently underwent hysterectomy due to abnormal uterine bleeding. Detailed gynecologic examinations, patient histories, and reports of endometrial sampling and hysterectomy were recorded. Cohen’s Kappa (κ) statistic was utilized to evaluate the concordance between histopathological results from an endometrial biopsy and hysterectomy. **Results:** The mean age of the cohort was 47 ± 4 years. Endometrial biopsies predominantly revealed benign findings, with 137 (40.4%) cases showing proliferative endometrium and 2 (0.6%) cases showing endometrial cancer. Following hysterectomy, final pathology indicated proliferative endometrium in 208 (61.4%) cases, with 7 (2.1%) cases showing endometrioid cancer. There was a statistically significant but low level of concordance between histopathological reports of endometrial biopsy and hysterectomy results (Kappa = 0.108; *p* < 0.001). Significant differences were observed only in the body mass index of patients based on hysterectomy results (*p* = 0.004). When demographic characteristics were compared with cancer incidence, smoking status and preoperative endometrial biopsy findings showed statistically significant differences (*p* = 0.042 and *p* = 0.010, respectively). **Conclusions:** The concordance between the pathological findings of a preoperative endometrial biopsy and hysterectomy is low. Body mass index is an important differentiating factor between benign histopathologic findings of endometrium and endometrial neoplasia. Moreover, adenomyosis was found to be associated with endometrial cancer cases. The current approach to premenopausal women with abnormal uterine bleeding, which includes a routine endometrial biopsy, warrants re-evaluation by international societies and experts.

## 1. Introduction

Abnormal uterine bleeding refers to irregular bleeding from the uterine corpus that deviates from normal patterns in terms of regularity, volume, frequency, or duration in the absence of pregnancy [1]. It affects up to 30% of women during the reproductive stage. According to the updated The International Federation of Gynecology and Obstetrics (FIGO) classification, the etiology is categorized into structural and non-structural causes [1]. These include polyp, adenomyosis, leiomyoma, and malignancy for structural causes, and coagulopathy, ovulatory dysfunction, endometrial, iatrogenic, and not otherwise classified for non-structural causes [1]. According to the opinion of FIGO and other associations, endometrial sampling is recommended for patients experiencing abnormal uterine bleeding above the age of 40 or 45 before considering them as having a low risk profile in terms of malignant neoplasms [1,2,3].

Endometrial sampling allows practitioners to promptly diagnose endometrial cancer by the most common symptom: abnormal uterine bleeding (observed in 90% of patients) [4]. Given that the 5-year survival rate at stage 1 is approximately 80%, early diagnosis is of the utmost importance for improving survival rates [5]. The studies so far have indicated that preoperative endometrial sampling serves as a moderate predictor of endometrial cancer, but it may not accurately predict the spread and recurrence risk of the disease [6]. Given that the prevalence of endometrial cancer among premenopausal women is relatively low compared to postmenopausal women with postmenopausal bleeding, accurate predictive models should be developed to direct patients to endometrial sampling and further investigation [7,8]. Moreover, it is crucial that the pathology report from preoperative endometrial sampling corresponds with the final pathological report of the hysterectomy specimen to ensure proper patient management.

The most important predictors of endometrial malignancy are age, parity, obesity, endometrial thickness, individual and genetic risk factors, and family history [9,10,11,12]. In symptomatic premenopausal women, a body mass index >30 kg/m^2^ is reported as a significant risk factor for endometrial neoplasia [12]. A first-degree family history of endometrial cancer carries a 1.82 relative risk of developing the disease [11]. Since preoperative endometrial sampling is an invasive procedure associated with potential complications such as infection and bleeding, as well as imposing financial burdens and leading to loss of time and labor force [13], the decision to perform endometrial sampling should be based on individualized assessments of patient risks.

Given the low incidence of endometrial cancer in premenopausal women with abnormal uterine bleeding, which can be caused by various factors as listed above, routine endometrial sampling is neither feasible nor cost-effective. However, in routine clinical practice, both international guidelines and the fear of missing a malignancy lead practitioners to perform endometrial sampling in each case of abnormal uterine bleeding. In this study, we aimed to evaluate the necessity and usefulness of preoperative endometrial sampling by assessing premenopausal women who underwent hysterectomy for abnormal uterine bleeding after preoperative endometrial sampling at our clinic.

## 2. Materials and Methods

In this retrospective cohort study, we analyzed 477 premenopausal women who underwent hysterectomy for abnormal uterine bleeding at the Department of Obstetrics and Gynecology of Istanbul University-Cerrahpasa, Cerrahpasa Faculty of Medicine, between 2015 and 2023. We included women who underwent hysterectomy for abnormal uterine bleeding after preoperative endometrial sampling. The causes of abnormal uterine bleeding were defined according to the updated FIGO classification [1]. We included patients with abnormal uterine bleeding due to both structural causes and non-structural causes (ovulatory dysfunction and endometrial). We excluded women who underwent hysterectomy for other gynecological malignancies and those whose abnormal uterine bleeding was due to coagulopathy, iatrogenic causes, or unclassified causes. Detailed gynecologic examinations and patient histories (including age, body mass index (BMI), gravidity, parity, menstrual history, systemic diseases and medication, therapeutic history of the patient for abnormal uterine bleeding, family history) were recorded from the electronic database. In addition, reports of both endometrial sampling and hysterectomy, hemoglobin and hematocrit levels, and need for transfusion were extracted from the electronic health record.

All procedures performed in studies involving human participants were in accordance with the 1964 Helsinki Declaration and its later amendments or comparable ethical standards. Approval was granted by the institutional review board (approval date: 2 June 2023 and approval number: 701661). Informed consent was obtained from all patients in compliance with the Declaration of Helsinki.

### Statistical Analysis

Post hoc power analysis was performed with the ClinCalc online post hoc power calculator. This calculator uses various equations to calculate the statistical power of a study after the study is performed. The study had 99.2% power to produce a significant difference with patients in terms of BMI for hysterectomy results. 

The normality of continuous variables was investigated with Shapiro–Wilk’s test. Descriptive statistics were presented using mean and standard deviation for normally distributed variables and median (minimum–maximum) for the non-normally distributed variables.

Non-parametric statistical methods were used for values with skewed distribution. For the comparison of two independent non-normally distributed groups, the Mann–Whitney U test was used. For the comparison of more than two independent non-normally distributed groups, the Kruskal–Wallis test was used. For the comparison of two dependent non-normally distributed groups, the Wilcoxon test was used.

Cohen’s Kappa (κ) is used to measure inter-rater reliability to assess the agreement between two or more methods when categorizing items into multiple categories.

The χ^2^ (Fisher’s Exact if necessary) test was used for categorical variables and expressed as observation counts (and percentages).

Statistical significance was accepted when the two-sided *p* value was lower than 0.05. SPSS version 26.0 (SPSS Science, Chicago, IL, USA) was used for the statistical analysis of the presented data.

## 3. Results

Of the 477 premenopausal women who underwent hysterectomy for abnormal uterine bleeding, only 339 had preoperative endometrial sampling and were eligible according to our criteria. Thus, only these 339 were included in the final analysis.

### 3.1. The Demographic Findings

The demographic characteristics of our cohort are presented in Table 1.

The mean age of our cohort was 47 ± 4 and the mean BMI value was 29.89 ± 5.41 kg/m^2^. Of these women, 82 (24.4%) were smokers, 147 (43.6%) had accompanying comorbidities such as hypertension and cardiovascular disease, and 40 had diabetes. Only 2 were receiving hormonal treatment for menopausal symptoms and endometriosis, while 114 were undergoing treatment for abnormal uterine bleeding, mostly with a levonorgestrel-releasing intrauterine device. Upon ultrasound examination, the most common organic pathology observed was myoma uteri, followed by adenomyosis. Histopathologic analysis of endometrial biopsy specimens revealed predominantly benign findings: 137 (40.4%) had proliferative endometrium, 126 (37.2%) had endometrial polyp, 30 (8.8%) had non-atypical hyperplasia, 18 (5.3%) had secretory/iatrogenic endometrium, and 22 (6.5%) had atypical hyperplasia. Only two (0.6%) patients were diagnosed with endometrial cancer based on the biopsy results.

### 3.2. The Histopathology Results

The final histopathology reports of hysterectomy revealed proliferative endometrium in 208 (61.4%) cases and secretory endometrium in 20 (5.9%) cases. Atypical hyperplasia was slightly more common than non-atypical hyperplasia, with 12 (3.5%) cases compared to 10 (2.9%) cases, respectively (Table 2).

According to the final pathology, 10 (2.9%) patients were diagnosed with cancer, including 1 case of ovarian cancer. Of these, seven (2.1%) were diagnosed with endometrioid cancer, while two (0.6%) were diagnosed with sarcoma, and one (0.3%) was diagnosed with a smooth muscle tumor of uncertain potential (STUMP). The preoperative endometrial biopsy results for the seven endometrial cancer cases were as follows: five patients had atypical hyperplasia and two had cancer. Among these cases, four were classified as stage 1a, two as stage 1b, and one as stage 2. One patient initially diagnosed with Grade 1 endometrial adenocarcinoma based on a preoperative biopsy was later reclassified as Grade 2 according to her hysterectomy result. None of the endometrial cancer cases required additional surgery. For the sarcoma cases, none of the endometrial biopsy results reported cancer. The hysterectomy results revealed adenomyosis accompanying the endometrial pathologies in 41.0% of the cases. Moreover, one of the endometrial cancer patients had accompanying adenomyosis, whereas only one of the sarcoma patients had accompanying adenomyosis. In the same surgical session, 248 (73.2%) patients underwent oophorectomy.

There was a statistically significant but low level of compatibility between the histopathology results of an endometrial biopsy and hysterectomy (Kappa = 0.108; *p* < 0.001) (Table 3).

Table 4 presents a comparison of demographic characteristics according to the hysterectomy results. The only statistically significant difference observed was in BMI among the patients (*p* = 0.004). The mean value of BMI was higher in non-atypical hyperplasia, atypical hyperplasia, and cancer cases. There was no difference in terms of gravidity, parity, age, preoperative endometrial thickness, smoking status, diabetes mellitus, family history of cancer, accompanying adenomyosis, or other chronic diseases.

In relation to cancer, smoking status and preoperative endometrial biopsy findings were found to be statistically significantly different compared to the others (*p* = 0.042 and *p* = 0.010, respectively) (Table 5).

## 4. Discussion

In this retrospective study conducted among premenopausal women who underwent hysterectomy due to abnormal uterine bleeding, we found that the compatibility between the pathological findings of the preoperative endometrial biopsy and hysterectomy was rather low. We observed an association between the final diagnosis of endometrial cancer and atypical hyperplasia and/or cancer in the preoperative endometrial biopsy, while such an association was absent for sarcoma cases. We confirmed that BMI is an important differentiating factor between benign histopathologic findings of endometrium and endometrial neoplasia. Moreover, adenomyosis was found to be associated with endometrial cancer cases. The most common histopathological diagnosis in premenopausal women with abnormal uterine bleeding in our study was proliferative endometrium, followed by endometrial polyp. This differs from postmenopausal cases of abnormal bleeding, where endometrial atrophy has been reported to be the most common pathological finding, followed by endometrial polyp [14].

An endometrial biopsy, which is commonly used for diagnosing endometrial cancer, should be performed based on individual risk factors. Patients with postmenopausal bleeding and thickened endometrium during menopause are considered good candidates for an endometrial biopsy due to their elevated risk of endometrial cancer. However, for premenopausal individuals, current recommendations and algorithms do not adequately assess underlying risks and guide patient management [15]. Our study emphasizes the high rate of benign histopathologic findings of endometrium observed in preoperative samplings, suggesting that obtaining an endometrial biopsy before hysterectomy for abnormal uterine bleeding may not always be necessary. While we acknowledge the necessity of large-scale multicenter clinical studies to develop valid risk prediction models and algorithms, we do not recommend a routine endometrial biopsy prior to each hysterectomy in daily practice.

The diagnostic approach should be individualized based on factors such as family history, comorbidities, pelvic exam, history of hormone usage, and history of cancer [16]. In our study, we found no difference in terms of comorbidities, family history of cancer, preoperative endometrial thickness, age, or parity when we compared the cases with benign pathology, hyperplasia, and cancer. We confirmed that BMI and preoperative endometrial sampling results are significant for distinguishing cancer from other pathologies, as suggested by Verbakel et al. [10]. According to our findings, in obese premenopausal women, the hysterectomy pathology material was statistically significantly associated with premalignant and malignant endometrial neoplasia. In this regard, we confirmed that obesity is a significant risk factor for endometrial neoplasia [17]. Despite our single-center results, we may recommend preoperative endometrial sampling before planned surgery in premenopausal women with a BMI above 30 kg/m^2^. The American Institute for Cancer Research reported a 50% increase in the risk of developing endometrial cancer for obese women [18], and obesity is especially associated with aggressive subtypes of endometrial cancer [19]. Wise et al. also showed that premenopausal women with a BMI above 30 kg/m^2^ are four times more likely to develop endometrial hyperplasia or cancer [12]. Unopposed estrogen exposure raises the risk of endometrial cancer [20]. In premenopausal obese women, this results from progesterone deficiency due to anovulation and increased estrogen production due to increased adiposity [21]. In the absence of progesterone to oppose estrogen’s proliferative effects, endometrial hyperplasia occurs. Moreover, we found that the incidence of malignancy is low (2.9%) in the premenopausal state, with proliferative endometrium being the most common histopathologic result of endometrial sampling (61.4%), consistent with previous studies [22,23,24]. As a tertiary referral hospital, we observed a relatively high rate of cancer compared to other studies where the cancer rate ranged between 0.12 and 1.2% among patients undergoing hysterectomies for benign indications [15,22,25,26,27]. Additionally, we found that adenomyosis accompanied 25% of our endometrial cancer cases, in line with the literature [28,29]. It has been suggested that endometrial cancer coexisting with adenomyosis has a low histologic tumor grade, low FIGO stage, and better prognosis [30,31]. Although the exact mechanism cannot be explained, the neoplastic potential in adenomyotic patients may be due to common risk factors such as hyperestrogenism (high BMI, hormone treatment, etc.), genetic mutations, and inflammatory factors that stimulate angiogenesis and cell proliferation [28,30]. Therefore, we suggest that adenomyosis screening could be added as a new risk parameter to the guidelines.

The endometrial biopsy, while commonly used for diagnosing endometrial cancer, is a procedure associated with several morbidities, such as pain, infection, uterine perforation, and bleeding [13,32,33,34]. Even though it may seem like a minimally invasive, easy-to-perform, low-cost procedure, it does not always provide reliable results or advantages for diagnosing premalignant and malignant lesions when performed on large numbers of patients. Additionally, it may impose financial burdens and lead to loss of time and labor force. We did not observe any major complications in our patients, and we did not calculate the financial cost since, in our country, health expenses are covered by the government. Since their results do not provide specific guidance for patient management, endometrial biopsies should be individualized based on risk factors rather than performed routinely on every patient.

The primary limitation of our study is its single-center retrospective design and small sample size, which prevents the generalization of our results to the broader population. However, it has high power according to post hoc power analysis. Another limitation is the inconsistency between endometrial thickness values and pathology results. In the literature, endometrial thickness is reported to be associated with benign, premalignant, and malignant lesions of the uterus, with a thicker endometrium indicating a higher risk for malignancy [14]. This discrepancy may be due to the lack of standardized endometrial thickness measurements across the menstrual cycle. Furthermore, our inclusion of only premenopausal patients may have limited our ability to assess the impact of age on endometrial cancer rates. In addition, an important strength of our study is the consistency in pathological reporting by the same professor for each specimen, minimizing interobserver variability in pathological reports.

## 5. Conclusions

In conclusion, the compatibility between preoperative endometrial biopsy findings and hysterectomy results is low. Obesity and adenomyosis have been found to be associated with endometrial neoplasia. Offering endometrial sampling to every premenopausal woman with abnormal uterine bleeding before hysterectomy may result in a high rate of benign pathologies in preoperative samplings. Therefore, clear algorithms or valid risk prediction models are needed to accurately assess the risk of endometrial cancer and prevent unnecessary endometrial biopsies in premenopausal women. The current approach to premenopausal women with abnormal uterine bleeding, which includes a routine endometrial biopsy, is contentious and warrants reconsideration by international societies and experts.

## Figures and Tables

**Table 1:** The demographic characteristics of the cohort.

		Mean (SD) (N = 339)	Med (Min–Max) (N = 339)
Age		47 (4)	47 (34–62)
Body mass index (BMI) (kg/m^2^)		29.89 (5.41)	29.2 (18.4–44.9)
Endometrial thickness (mm)		9.41 (5.17)	8.55 (2–31.3)
Preoperative hemoglobin (g/dL)		11.26 (2.18)	11.8 (3.6–15.4)
Preoperative hematocrit (%)		34.3 (6.02)	35.05 (11.4–46.2)
Postoperative hemoglobin (g/dL)		10.2 (1.51)	10.3 (6.6–14.8)
Postoperative hematocrit (%)		31 (4.22)	31.4 (20.8–44)
		**n/N (%)**	
Smoking		82/336 (24.4)	
Presence of comorbidity		147/337 (43.6)	
Presence of diabetes		40/337 (11.9)	
On hormonal treatment		2/338 (0.6)	
Presence of family history of cancer		16/269 (5.9)	
Treatment for abnormal uterine bleeding	None	168/282 (59.6)	
	Type unknown	56/282 (19.9)	
	MIRENA	38/282 (13.5)	
	Oral progesterone	18/282(6.4)	
	Oral contraceptive	2/282 (0.7)	
Uterine organic pathology	Myoma uteri	198/339 (58.4)	
	None	78/339 (23.0)	
	Adenomyosis	52/339 (15.3)	
	Polyp	11/339 (3.2)	
Endometrial biopsy result	Proliferative endometrium	137/339 (40.4)	
	Polyp	126/339 (37.2)	
	Non-atypical hyperplasia	30/339 (8.8)	
	Atypical hyperplasia	22/339 (6.5)	
	Secretory/iatrogenic endometrium	18/339 (5.3)	
	Atrophic	3/339 (0.9)	
	Cancer	2/339 (0.6)	
	Placental site nodule	1/339 (0.3)	

**Table 2:** The final histopathology reports of hysterectomy.

		n	(%)
Hysterectomy report (N = 339)	Proliferative endometrium	208	61.4
	Polyp	63	18.6
	Secretory–iatrogenic	20	5.9
	Atrophy	15	4.4
	Atypical hyperplasia	12	3.5
	Cancer	10	2.9
	Non-atypical hyperplasia	10	2.9
	Extrauterine cancer	1	0.3
Cancer type	Endometrioid cancer	7	2.1
	Sarcoma	2	0.6
	Stump	1	0.3
	Ovarian serous borderline tumor	1	0.3
Presence of adenomyosis	Absent	200	59.0
	Present	139	41.0
Oophorectomy	No	91	26.8
	Yes	248	73.2

**Table 3:** The compatibility between the pathological findings of preoperative endometrial biopsy and hysterectomy.

	Hysterectomy Report																
Endometrial Biopsy Report		**Proliferative Endometrium**		**Secretory–Iatrogenic Endometrium**		**Non-Atypical Hyperplasia**		**Atypical Hyperplasia**		**Polyp**		**Atrophic**		**Cancer**		**Extrauterine Cancer**	
		**N**	**%**	**N**	**%**	**N**	**%**	**N**	**%**	**N**	**%**	**N**	**%**	**N**	**%**	**N**	**%**
	Proliferative endometrium	94	45.2	8	40.0	1	10.0	0	0	26	41.3	7	46.7	1	10.0	0	0
	Secretory–iatrogenic endometrium	10	4.8	3	15.0	0	0	0	0	3	4.8	1	6.7	1	10.0	0	0
	Non-atypical hyperplasia	12	5.8	3	15.0	4	40.0	0	0	10	15.9	1	6.7	0	0	0	0
	Atypical hyperplasia	4	1.9	0	0	3	30.0	9	75.0	1	1.6	0	0	5	50.0	0	0
	Polyp	86	41.3	6	30.0	2	20.0	3	25.0	22	34.9	5	33.3	1	10.0	1	100.0
	Atrophic	1	0.5	0	0	0	0	0	0	1	1.6	1	6.7	0	0	0	0
	Cancer	0	0	0	0	0	0	0	0	0	0	0	0	2	20.0	0	0
	Placental site nodule	1	0.5	0	0	0	0	0	0	0	0	0	0	0	0	0	0

**Table 4:** The comparison of demographic characteristics according to hysterectomy results.

	Benign	Non-Atypical Hyperplasia		Atypical Hyperplasia			Cancer			*p*
	Mean (SD) Median (Min–Max)	Mean (SD) Median (Min–Max)		Mean (SD) Median (Min–Max)			Mean (SD) Median (Min–Max)			
Age	47 (4)	47 (5)		48 (6)			50 (7)			0.200
	47 (34–56)	46 (39–55)		50 (36–60)			51 (38–62)			
Gravidity	3.55 (1.99)	3.5 (1.78)		4.08 (1.78)			3.56 (3.05)			0.700
	3 (0–13)	4 (1–7)		4 (2–7)			3 (0–9)			
Parity	2.87 (1.92)	3 (1.49)		2.58 (1.16)			2.33 (2.06)			0.596
	3 (0–21)	3 (1–5)		2.5 (1–5)			2 (0–7)			
Body mass index (BMI)	29.33 (5.35)	35.71 (4.48)		34.13 (5.71)			33.08 (6.16)			**0.004**
	28.95 (19.1–44.9)	34.87 (31.2–41.9)		34.9 (26.1–43.82)			36.1 (23.4–39)			
Preoperative endometrial thickness (mm)	9.08 (4.94)	10.58 (5.55)		11.99 (5.71)			8.31 (2.09)			0.256
	8.4 (2–31)	8.1 (4.6–20)		10.5 (5.45–24)			8.3 (5–11)			
		**n**	**%**	**n**	**%**	**n**	**%**	**n**	**%**	***p***
Smoking status	No	152	73.4	9	90.0	9	75.0	4	44.4	0.181
	Yes	55	26.6	1	10.0	3	25.0	5	55.6	
Diabetes mellitus	No	187	90.3	10	100.0	11	91.7	8	88.9	0.876
	Yes	20	9.7	0	0	1	8.3	1	11.1	
Family history of cancer	No	156	94.0	7	100.0	11	100.0	7	87.5	0.658
	Yes	10	6.0	0	0	0	0	1	12.5	
Accompanying adenomyosis	No	126	60.6	6	60.0	3	25.0	8	80.0	0.056
	Yes	82	39.4	4	40.0	9	75.0	2	20.0	
Other chronic disease	No	119	57.5	3	30.0	7	58.3	3	33.3	0.200
	Yes	88	42.5	7	70.0	5	41.7	6	66.7	

Bold values denote statistical significance at the *p* < 0.05 level.

**Table 5:** Comparison of the demographic findings in relation to cancer.

		Others		Cancer		*p*
		N	%	N	%	
Smoking	No	250	76.5	4	44.4	**0.042**
	Yes	77	23.5	5	55.6	
Diabetes mellitus	No	289	88.1	8	88.9	0.710
	Yes	39	11.9	1	11.1	
Family history of cancer	No	246	94.3	7	87.5	0.392
	Yes	15	5.7	1	12.5	
Accompanying adenomyosis	No	192	58.4	8	80.0	0.148
	Yes	137	41.6	2	20.0	
Other chronic disease	No	187	57.0	3	33.3	0.142
	Yes	141	43.0	6	66.7	
Pathological findings of preoperative endometrial biopsy	Proliferative endometrium	136	41.3	1	10.0	**0.010**
	Secretory–iatrogenic endometrium	17	5.2	1	10.0	
	Non-atypical hyperplasia	30	9.1	0	0	
	Atypical hyperplasia	17	5.2	5	50.0	
	Polyp	125	38.0	1	10.0	
	Atrophic	3	0.9	0	0	
	Cancer	0	0	2	20.0	
	Placental site nodule	1	0.3	0	0	
		**Mean (SD)** **Median (min–max)**		**Mean (SD)** **Median (min–max)**		***p***
Gravidity		3.67 (2.22)		3.56 (3.05)		0.744
		3 (0–17)		3 (0–9)		
Parity		2.9 (2.01)		2.33 (2.06)		0.255
		2.5 (0–21)		2 (0–7)		
Age		47 (4)		50 (7)		0.130
		47 (34–60)		51 (38–62)		
Body mass index (kg/m^2^)		29.78 (5.37)		33.08 (6.16)		0.131
		29.1 (18.4–44.9)		36.1 (23.4–39)		
Preoperative endometrial thickness (mm)		9.44 (5.23)		8.31 (2.09)		0.813
		8.6 (2–31.3)		8.3 (5–11)		

Bold values denote statistical significance at the *p* < 0.05 level.

## Data Availability

The raw data of this article will be sent to anyone by the first author, without undue reservation.
